# BioFed: federated query processing over life sciences linked open data

**DOI:** 10.1186/s13326-017-0118-0

**Published:** 2017-03-15

**Authors:** Ali Hasnain, Qaiser Mehmood, Syeda Sana e Zainab, Muhammad Saleem, Claude Warren, Durre Zehra, Stefan Decker, Dietrich Rebholz-Schuhmann

**Affiliations:** 1 Insight Centre for Data Analytics, National University of Ireland (NUIG), Galway, Ireland; 2 Universität Leipzig, IFI/AKSW, Leipzig, PO 100920, D-04009 Germany; 3 IBM, IDA Business Park, Galway, Ireland

**Keywords:** Life sciences dataset, Linked open data, SPARQL query federation

## Abstract

**Background:**

Biomedical data, e.g. from knowledge bases and ontologies, is increasingly made available following open linked data principles, at best as RDF triple data. This is a necessary step towards unified access to biological data sets, but this still requires solutions to query multiple endpoints for their heterogeneous data to eventually retrieve all the meaningful information. Suggested solutions are based on query federation approaches, which require the submission of SPARQL queries to endpoints. Due to the size and complexity of available data, these solutions have to be optimised for efficient retrieval times and for users in life sciences research. Last but not least, over time, the reliability of data resources in terms of access and quality have to be monitored. Our solution (BioFed) federates data over 130 SPARQL endpoints in life sciences and tailors query submission according to the provenance information. BioFed has been evaluated against the state of the art solution FedX and forms an important benchmark for the life science domain.

**Methods:**

The efficient cataloguing approach of the federated query processing system *’BioFed’*, the triple pattern wise source selection and the semantic source normalisation forms the core to our solution. It gathers and integrates data from newly identified public endpoints for federated access. Basic provenance information is linked to the retrieved data. Last but not least, BioFed makes use of the latest SPARQL standard (i.e., 1.1) to leverage the full benefits for query federation. The evaluation is based on 10 simple and 10 complex queries, which address data in 10 major and very popular data sources (e.g., Dugbank, Sider).

**Results:**

BioFed is a solution for a single-point-of-access for a large number of SPARQL endpoints providing life science data. It facilitates efficient query generation for data access and provides basic provenance information in combination with the retrieved data. BioFed fully supports SPARQL 1.1 and gives access to the endpoint’s availability based on the EndpointData graph. Our evaluation of BioFed against FedX is based on 20 heterogeneous federated SPARQL queries and shows competitive execution performance in comparison to FedX, which can be attributed to the provision of provenance information for the source selection.

**Conclusion:**

Developing and testing federated query engines for life sciences data is still a challenging task. According to our findings, it is advantageous to optimise the source selection. The cataloguing of SPARQL endpoints, including type and property indexing, leads to efficient querying of data resources over the Web of Data. This could even be further improved through the use of ontologies, e.g., for abstract normalisation of query terms.

## Background

The Web provides access to large-scale sets of interlinked data from heterogeneous scientific domains, and – in particular for the life science researchers – develops into a source of reference data from scientific experiments [1]. The comprehensive set of Linked Open Data (LOD)^1^ covers over 60 billion triples provided by more than 1’000 different data sets. The small but important portion of the Linked Open Data cloud is composed of the Life Science Linked Open Data (LS-LOD), which results to 8% (83 data sets) of the overall LOD cloud^2^. The life science data contributes significantly to the ongoing research in semantic Web technologies, since the life science research community gathers and exposes their expertise in form of high quality ontologies, which support innovative retrieval methods across distributed SPARQL endpoint engines as presented in this publication.

Significant contributions in terms of data integration and data provision have been made available from the Bio2RDF project^3^, the Linked Life Data initiative^4^, the Neurocommons group^5^, through the Healthcare and Life Sciences knowledge base^6^ (HCLS Kb), from the Linked Cancer Genome Atlas (Linked TCGA) [2, 3], and the W3C HCLSIG Linking Open Drug Data (LODD) initiative^7^. The outcomes from these initiatives develop themselves into reference data sources that feed the existing life science expertise back into the ongoing large-scale research, for example into high-throughput gene sequencing research with a need to access the full body of biomedical data [4]. As a natural consequence, a single point of access and reference to the life sciences (LS) data is an important step forward in using the data and – eventually – in mastering the data deluge.

It has already been an important step forward to integrate and RDF-ize the existing biological knowledge sources to make the data available according to semantic Web and open data principles, but this is not sufficient for efficient data access. Further improvements have to deal with the access to the existing SPARQL endpoints, access to the meta-data of the data repositories, balancing access overheads against query efficiency and ultimately, the efficient use of all technological advancements altogether [5]. The resulting solution should cope with the size and the complexity of the data, and should still provide full access to the data in a way that a researcher can formulate and explore complex queries in reference to the full amount of integrated data without large processing overheads, i.e. the the heterogeneity of the data should not impair the identification of meaningful results [6–8]. In particular, the exploitation of reference meta-data information and the use of state of the art federation technologies form an important step for the evaluation of such an engine in a real-life use case scenario.

The integration of heterogeneous data sources takes place in research teams that make the result available as a SPARQL endpoint, leading to the challenge of combining disparate SPARQL endpoints with the help of federation engines, which a priori rely on the federation of queries being delivered across the distributed resources [9]. The latest SPARQL standard, i.e. SPARQL 1.1, is a key technological advancement to assemble federated queries (with the help of the “SERVICE” query option), and is supported by SWobjects^8^, Apache Jena^9^ and dotNetRDF^10^. This resulted into the development of different systems [10–15] capable of executing queries in a federated environment and claiming that this approach is sufficiently generic for processing federated queries over any other data set. However, specific draw-backs have to be considered that will be addressed in the presented solution:

- First, the federation of queries does not enforce that the queries deliver the expected results, i.e. access to meta-data information from the SPARQL endpoints should improve the outcomes.
- Second, preparing meaningful and productive SPARQL queries remains to form a skillful task and profits from domain expertise (e.g., from the domain ontologies) as well as the meta-data information from the data sources.
- Last, once the meta-data information has been used to define the query (to be federated across endpoints), optimisations solutions should apply to enable efficient, i.e. speedy, response times.

BioFed is a federated query engine that makes use of state of the art semantic Web technologies to query large and heterogeneous data sets in the life sciences domain: the federation covers 130 public SPARQL endpoints optimised for LS-LOD. It offers a single-point-of-access for distributed LS data enabling scientists to access the data from reliable sources without extensive expertise in SPARQL query formulation (for SPARQL 1.1, online user interface with drop down menus). Its autonomous resource discovery approach identifies relevant triple patterns, matches types according to their labels as a basic semantic normalisation approach, and optimises the retrieval based on source selection strategies for efficient response times. New public endpoints are added through a cataloguing mechanism based on source selection [16]. The provided provenance information covers *the sources queried*, *the number of triples returned* and *the retrieval time*.

The remaining part of this paper is organised as follows: we present related work in Section “Related work”. Then we present the methodologies covering the implementation details including discovery, source selection and query re-writing (Section “Methods”). BioFed salient features are presented in Section “BioFed salient features”. The results and the evaluation against the query engine FedX is given in Section “Results and discussion”. Section “Conclusions” covers the conclusion, discussion and future work.

## Related work

Advances in federated query processing methods form the key achievement for federated query engines that automatically access data from multiple endpoints. Each of the suggested solutions follows slightly different principles and even goals, and realises different trade-offs between speed, completeness, and flexibility requirements, which are partially imposed by the status of technological advancements at that time the data sources ready for use.

Umbrich et al. [17, 18] proposed – in a straight forward way – a Qtree-based index structure that summarises the content of data sources for query execution over the Web of Data. The index gives access to the data in the SPARQL endpoint, but comes with significant draw-backs such as a lack of access to relational information, e.g., from the meta-data of the SPARQL endpoint, the overheads in pre-processing the existing data, and the consequence of out-of-date indexes and index rebuilding needs.

In terms of advanced index assisted approaches, the SHARE project registry [19] stores the index information as OWL class definitions and instances of the myGrid ontology. Similarly, OpenLifeData [20], indexed Bio2RDF using its semantically rich entity-relationships and exposed it as SADI services after registering in the SHARE registry. SHARE project stores the set of distinct predicates for all the endpoints. The source selection is performed by matching the predicate of the triple pattern against the set of predicates of all indexed endpoints. All the endpoints which contain matching predicates are selected as relevant sources for that triple pattern.

Kaoudi et al. [21] propose a federated query technique on top of distributed hash tables (DHT), which is a similar approach to the indexing techniques used by Umbrich et al. The DHT-based optimiser makes use of three greedy optimisation algorithms for best plan selection. Overall, the authors achieve good query execution times, but suffer from the same disadvantages as the previous solution.

Avalanche [22] gathers endpoint data sets statistics and bandwidth availability on-the-fly before the query federation, which increases the overall query execution time. Vandervalk et al. [23], presented two approaches for query optimisation in a distributed environment, requiring basic statistics regarding RDF predicates to query the remote SPARQL endpoints. For one approach a static query plan is computed in advance of query execution, using graph algorithms for finding minimum spanning trees. Whereas, in the second approach, the planning and execution of the query are evaluated to follow an independent query plan.

Quilitz and Leser [24] have developed DARQ for the federation of queries across SPARQL endpoints. It optimises the selection of relevant data sources on the bases of data descriptions, e.g., usage of predicates in the endpoint, and statistical information, to optimise the routing of queries to associated endpoints. This approach is straight forward, but could exploit better the distribution of triples given from a specific data source.

Langegger et al. in [25] describe a similar solution using a mediator approach, which continuously monitors the SPARQL endpoints for any changes in the data sets and updates the service descriptions automatically. They solve the problem of out-of-date descriptions, but unfortunately the authors have introduced the restriction that all subjects of triple statements must be variables for the bound predicate requirement of DARQ.

Schwarte et al. [11] have build FedX, which is a query federation engine for the Web of Data and which does not require an index for accessing the distributed data. FedX makes use of SPARQL ASK queries to enquire about the content and to determine the endpoints with relevant information. This approach provides sufficiently fast data retrieval as compared to other prior art techniques [26], however, it under-exploits data provide from the endpoint up front to optimise the query generation.

Saleem et al. [13] presented DAW, a duplicate-aware federated query approach over the Web of Data. It makes use of the min-wise independent permutations [27] and compact data summaries to extend existing SPARQL query federation engines in order to achieve the same query recall values while querying fewer SAPRQL endpoints, which is a very specific optimisation solution for source selection. HiBISCuS [14] is an efficient hypergraph based source selection approach for SPARQL query federation over multiple SPARQL endpoints.

SPLENDID [26] exploits Vocabulary of Interlinked Datasets (VoID) descriptions that are provided from the SPARQL endpoints, and makes use of SPARQL ASK queries to determine relevant sources for the querying of specific triple patterns. This leads to the result that SPLENDID is able to federate more expressive queries in comparison to the previous solutions, but has not been tested on the very specific case of distributed SPARQL endpoints for the life sciences with their high complexity of data.

Other optimisation techniques have also been attempted. Li and Heflin [28] have built a tree structure that supports federated query processing over heterogeneous sources and uses a reasoner to answer queries over the selected sources and their corresponding ontologies. This approach offers new ways to use class specifications for complex querying, but has not been tested against challenging life science use cases either.

ELITE [29] is an entailment-based federated query processing engine. It makes use of the ontology-based data access, R-tree based indexing, query rewriting, and DL-Lite formalism to retrieve more complete results which other systems may miss due to no reasoning over given query.

Ludwig and Tran [30] propose a mixed query engine that assumes to encounter incomplete knowledge about the sources to select and discover new sources during run time, which would not scale sufficiently in the case of complex data and larger numbers of SPARQL endpoints. Acosta et al. [31] present ANAPSID, an adaptive query engine that adapts query execution schedulers to SPARQL endpoints data availability and run-time conditions, which would not scale to the life science domain either.

In BioFed we exploit the potential of VoID descriptors – the state of the art approach for describing any dataset in order to catalogue the classes and properties from remote SPARQL endpoints. This cataloguing mechanism facilitates query federation mechanism to access data from multiple heterogeneous biological datasources and offers the opportunity to support the user of the retrieval engine with efficient query formulation tools: the queries are build on the basis of existing data and then distributed to the relevant endpoints through the source selection approach. For this, BioFed adopts a hybrid source selection approach [1], i.e., we make use of both index and SPARQL ask queries.

Moreover BioFed covers the full range of public SPARQL endpoints in health care and life sciences domain, including Bio2RDF, which is a significant scope in terms of number of endpoints and complexity of data, and will remain to form a significant challenge for the semantic data integration of the near future. BioFed provides a single point of access for LS data with other important information e.g., provenance due to which some queries may take longer when compared to the other tools like FedX, whereas provenance is the key for the life sciences domain targeted by BioFed. One smaller-scale alternative approach is Topfed [3] which is a TCGA tailored federated query engine.

Furthermore, the information in the captured catalogue doesn’t rely on semantically rich entity-relationships, which would require complete knowledge of the defined schema, which – in return – is difficult to access for most of the used resources. Our focus is to cover a wide range of large-scale SPARQL endpoints and to catalogue sufficient information to achieve efficient querying of the federated resources.

It is worth noticing that the current interface provided by BioFed supports designing a basic set of SPARQL queries using a set of Query Elements (Qe) [16, 32, 33]. Different concepts and properties from endpoints acts as Qe in order to formulate SPARQL queries. Advanced and state of the Art query builders e.g., KnowledgeExplorer [34] and SPARQL Assist [35] make use of the original ontologies/vocabularies and provide an auto-complete mechanism to write a SPARQL query, but we believe BioFed interface is a step towards building a basic SPARQL query that queries over multiple LS SPARQL endpoints as BioFed offers the set of concepts and properties in a particular context that can easily be selected from drop-down menu in order to formulate SPARQL query.

## Methods

### General architecture

**Fig. 1:**
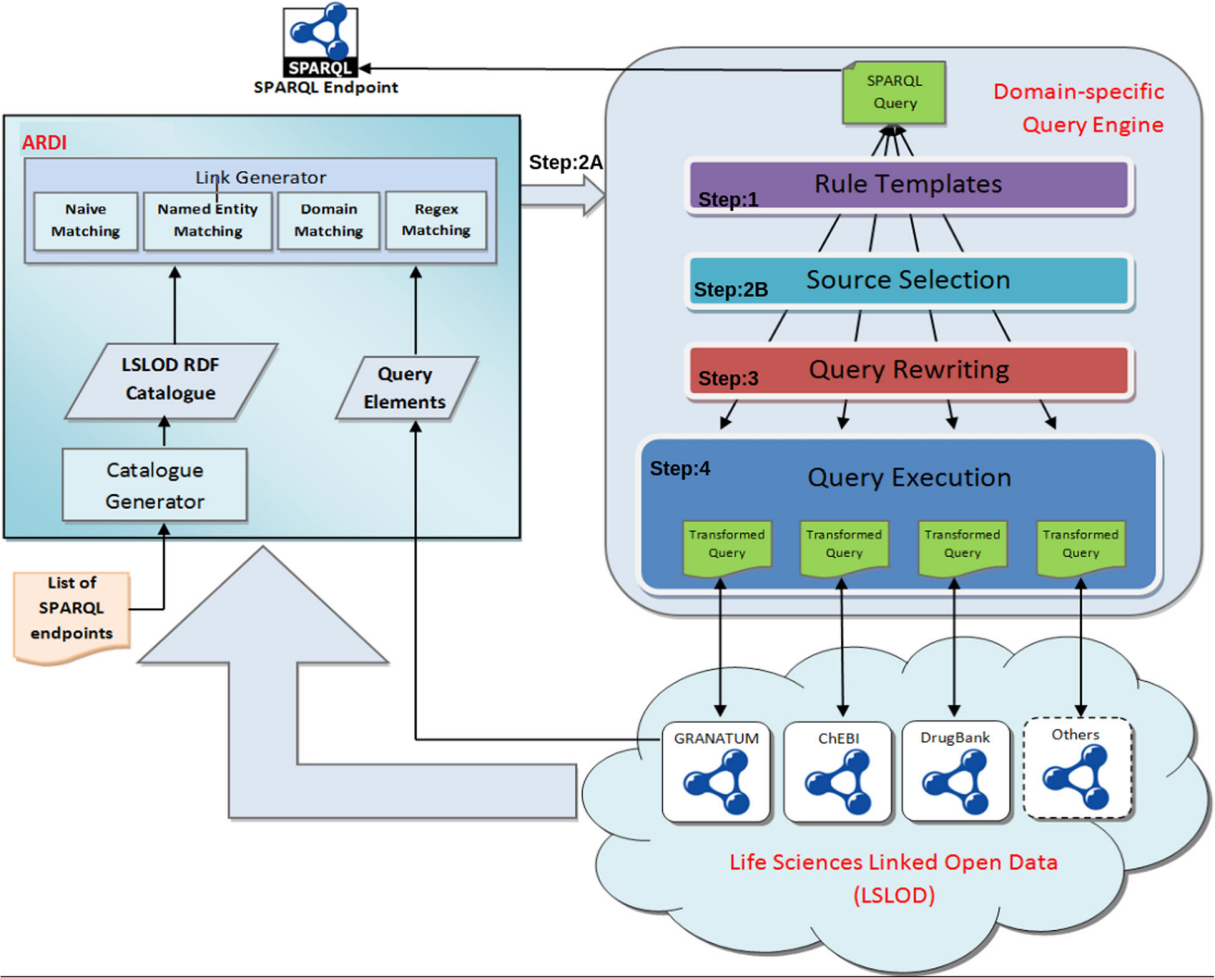
BioFed architecture. ARDI comes from previous work by Hasnain et al. [4, 16]

The general architecture of BioFed is given in Fig. 1. Given a SPARQL query, the first step is to parse the query and get the individual triple patterns (Step 1). The next step is the triple-pattern-wise source selection (TPWSS).

#### **Definition 1**

(***Total Triple Pattern-wise Sources Selected***) Let *Q* = {*t*_1_,…,*t*_*m*_} be a SPARQL query containing triple patterns *t*_1_,…,*t*_*m*_, $\mathcal {R}$ = $\{R_{t_{1}}, \ldots, R_{t_{m}} \}$ be the corresponding relevance set containing relevant data sources sets $R_{t_{1}}, \ldots, R_{t_{m}}$ for triple patterns *t*_1_,…,*t*_*m*_, respectively. We define TTPWSS = $\forall _{R_{t_{i}}\in \mathcal {R}} \, \sum |R_{t_{i}}| $ be the total triple pattern-wise sources selected for query Q, i.e., the sum of the magnitudes of relevant data sources sets over all individual triple patterns Q.

The TPWSS identify relevant (also called capable) sources against individual triple patterns of the query (Step 2B). BioFed performs this step by using the discovery approach presented in Hasnain et al. [16]. This discovery enumerates the known endpoints and relates each endpoint with one or more graphs and maps the local vocabulary to the vocabulary of the graph (Step2A). Step 3 is to convert the given SPARQL 1.0 query into corresponding SPARQL 1.1 query. This step is known as Query Re-writing and further explained below. BioFed makes use of the TPWSS information and the SPARQL “SERVICE” clause to rewrite the required SPARQL 1.1 query. The resulting SPARQL 1.1 query is executed on top of the Apache Jena query engine and the results are returned back (Step 4). In the following, we will explain each of these steps in detail.

BioFed is designed as a Domain Specific Query Engine (DSQE) that transforms expressions between existing vocabularies, i.e. for the vocabularies used by SPARQL endpoints, and combines those expressions into a single federated query using SPARQL “Service” calls. It does occur, that a single node is translated into multiple nodes (e.g., drug may become both a molecule and a smallMolecule) leading to multiple triples being created as a cross product from all possible node translations. The resulting statement is executed and then returns the results to the user. The algebra rewriter examines each segment of the BGP triples and attempts to expand the terms based on the vocabulary mapping into terms of the endpoint graphs and stores the result for each.

### Autonomous Resource Discovery and Indexing (ARDI)

The ARDI comprises a catalogue of LS-LOD and a set of functions to perform standard queries against it [4, 16]. The methodology for developing the ARDI consists of two stages namely *catalogue generation* and *link generation*. The methodology for catalogue generation relies on retrieving all “types” (distinct concepts) from each SPARQL endpoint and all associated properties with corresponding instances. URI patterns and example resources were also collected during this process. Data was retrieved from more than 130 public SPARQL endpoints^11^ and organised in an RDF document - the LS-LOD catalogue. The list of SPARQL endpoints was captured from publicly available Bio2RDF data sets and by searching for data sets in CKAN^12^ tagged *“life science”* or *“healthcare”*.

The methodology for link generation is presented in [16] where naïve, named entity and domain matching approaches for weaving the “types” together was discussed. We later extended our linking mechanism to incorporate “Regex Matching” [16].

BioFed utilises the ARDI to perform TPWSS. The ARDI is also used to determine the URLs of the SPARQL endpoints, and provides the base data for the endpoint selection logic. The ARDI examines each node in each triple in the BGP. For each triple it determines if there is a match in the ARDI or if the triple should be left unmatched (e.g., an RDF:type predicate). Each unmatched node is passed through unchanged.



The RDF in Listing 1 is an illustrative example of a portion of the catalogue generated for the KEGG SPARQL endpoint^13^. VoID is used for describing the data set and for linking it with the catalogue entries: the void#Dataset being described in this catalogue entry is “KEGG” SPARQL endpoint. In cases where SPARQL endpoints were available through mirrors (e.g., most Bio2RDF endpoints are available through Carleton Mirror URLs) or mentioned using alternative URLs (e.g., http://kegg.bio2rdf.org/sparql), these references were also added as a second value for the void#sparqlEndpoint property. ARDI extracts also includes one identified class (http://bio2rdf.org/ns/kegg#Enzyme), and couple of predicates including:

- (http://bio2rdf.org/ns/bio2rdf#url).
- (http://bio2rdf.org/ns/bio2rdf#synonym).
- (http://bio2rdf.org/ns/bio2rdf#isA).
- (http://bio2rdf.org/ns/kegg#systematicName).
- (http://bio2rdf.org/ns/kegg#xProduct).
- (http://bio2rdf.org/ns/kegg#xCofactor).
- (http://bio2rdf.org/ns/kegg#xSubstrate).
- (http://bio2rdf.org/ns/kegg#xGene).

Classes are linked to data sets using the void#class property; the labels were collected usually from parsing the last portion of the URI and probed instances were also recorded (http://bio2rdf.org/ec:3.2.1.161) as values for void#exampleResource. De-referencing the object URI <http://bio2rdf.org/cpd:C00001> resulted in the class <http://bio2rdf.org/kegg_resource:Compound>. We call this as a “range class” used as the range of the property <http://bio2rdf.org/ns/kegg#xSubstrate>. An actual object URI <http://bio2rdf.org/cpd:C00001> is classified as void#exampleResource of <http://bio2rdf.org/kegg_resource:Compound> and the URI regular expression pattern is recorded under void#uriRegexPattern. Whereas <http://bio2rdf.org/ns/kegg#Enzyme> is also regarded as “domain class”.

### Source selection

Like other SPARQL endpoint federation engines [11–14, 31], BioFed also performs triple pattern-wise source selection (TPWSS). The goal of the TPWSS is to identify the set of relevant (also called capable and is formally defined in [14]) data sources against individual triple patterns of the query. The reason behind TPWSS is to potentially ensure the result-set completeness of the federated SPARQL queries [14].

BioFed’s triple-pattern-wise source selection is shown in Algorithm 1 which takes the set of all available data sources ${\mathcal {D}}$, their ARDI/summaries ${\mathcal {S}}$, and a SPARQL query *Q* containing a set of triple patterns as input, and returns the set of relevant sources for individual triple patterns as output. It is a two step source selection algorithm: we first select relevant data sources for individual triple patterns (*lines 2–8*) and then prune the selected data sources in the second step (*lines 9–12*). Given a triple pattern *t*_*i*_∈*Q*, we initialise the relevant data source set $R_{t_{i}}$ to empty, and obtain the subject, predicate, and object of the triple pattern (*lines 3–4*). All data sources are selected as relevant for triple pattern with unbound predicate (*lines 5–6*). If the predicate *p* of a triple pattern *t*_*i*_ is bound(i.e., *p* is a URI) then we perform ARDI/summaries lookup for all data sources which contain the predicate *p* (*lines 7–8*). The relevant data source pruning step is performed for all triple patterns having a bound subject or a bound object (*line 9*). We send a SPARQL ASK query containing the triple pattern *t*_*i*_ to each of the relevant data source $r_{i} \in R_{t_{i}}\phantom {\dot {i}\!}$ and remove those data sources which fail the SPARQL ASK test, i.e., the SPARQL ASK query returns false (*lines 10–12*).

As an example, consider the query given in Listing 2. Starting from the first triple pattern <?drug drugbank:drugCategory drug-category:micronutrient>, predicate (i.e., drug bank:drugCategory) of the triple pattern is bound, thus a ARDI lookup will be performed. All data sources, which contain this predicate, will be selected as relevant for this triple pattern. In this example, DrugBank (the single relevant data source) will be selected. Since the object (i.e., drug-category:micronutrient) of the triple pattern is bound, a SPARQL ASK{?drug drugbank:drugCategory drug-category:micronutrient} query will be send to DrugBank to check whether it can provide results for the whole triple pattern. In this case, the SPARQL ASK query will result in true, thus the DrugBank will be finally selected as single relevant data source for the first triple pattern.

The second triple pattern only contains bound predicate and the ARDI lookup results in the DrugBank as the single relevant data source. It is important to note that both subject and object of the second triple pattern are unbound, thus no source pruning will be performed for this triple pattern.

Consider the third triple pattern, the predicate rdf:type is likely to be present in all data sources. Thus, the ARDI lookup will likely select all data sources as being relevant. However, since the object (i.e., kegg:Drug) of the triple pattern is bound, the data source pruning step will be performed: a SPARQL ASK{?keggDrug rdf:type kegg:Drug} query will be sent to all of the relevant data sources and only KEGG will be finally selected as the single relevant data source. The execution of the next two triple patterns are the same as the second triple pattern. KEGG is the only relevant data source for the fourth triple pattern while KEGG and ChEBI are relevant data sources for the fifth triple pattern.

### SPARQL 1.1 query re-writting

BioFed converts each SPARQL 1.0 query into corresponding SPARQL 1.1 query and executes it via the Jena API^14^. Before going into the details of re-writing SPARQL 1.1 query, we first introduce the notion of exclusive groups (used in SPARQL 1.1 query re-write) in the SPARQL query.

#### Exclusive groups

In a normal SPARQL query (i.e., not a federated query) execution, the user sends a complete query to the SPARQL endpoint and gets the results back from the endpoint, i.e., the complete query is executed at the SPARQL endpoint. Unlike normal SPARQL query execution, in general, the federated engine sends sub-queries to the corresponding SPARQL endpoints and gets the sub-query results back which are locally integrated by using different *join* techniques. The local execution of joins then results in high costs, in particular when intermediate results are large [11]. To minimise these costs, many of the existing SPARQL federation engines [11, 12] make use of the notion of Basic Graph Pattern (BGP) and Exclusive Groups (EG) which is formally defined as:

##### **Definition 2**

(***Basic Graph Pattern syntax***) The syntax of a SPARQL Basic Graph Pattern *BGP* expression is defined recursively as follows:

1. A tuple from (*I*∪*L*∪*V*∪*B*)×(*I*∪*V*)×(*I*∪*L*∪*V*∪*B*) is a graph pattern (a *triple pattern*).
2. The expressions (*P*_1_ AND *P*_2_), (*P*_1_ OPTIONAL *P*_2_) and (*P*_1_ UNION *P*_2_) are graph patterns, if *P*_1_ and *P*_2_ are graph patterns.
3. The expression (*P* FILTER *R*) is a graph pattern, if *P* is a graph pattern and *R* is a SPARQL constraint or filter expression.

##### **Definition 3**

Let BGP = { *t*_1_, …, *t*_*m*_} be a basic graph patterns (BGP)^15^ containing a set of triple patterns *t*_1_,…,*t*_*m*_, ${\mathcal {D}} = \{{D}_{1},\ldots, {D}_{n}\}$ be the set of distinct data sources, and $R_{t_{i}} = \{D_{1}, \ldots, D_{o}\} \subseteq {\mathcal {D}}$ be the set of relevant data sources for triple pattern *t*_*i*_. We define *E* *G*_*D*_={*t*_1_,…,*t*_*p*_}⊆*B* *G* *P* be the exclusive groups of triple patterns for a data soruce D $\in {\mathcal {D}}$ s.t. $\forall _{t_{i} \in EG_{D}} \, R_{t_{i}} = \{D\} $, i.e., the triple patterns whose single relevant source is D.

The advantage of exclusive groups (size greater than 1) is that they can be combined together (as a conjunctive query) and sent to the corresponding data source (i.e., SPARQL endpoints) in a single sub-query, thus considerably minimising: the number of remote requests, the number of irrelevant intermediate results, and the network traffic [11]. This is because in many cases the intermediate results of the individual triple patterns are often excluded after performing the join between the intermediary results of another triple pattern in the same. On the other hand, the triple pattern joins in the exclusive groups are directly performed by the data source itself, thus all intermediary irrelevant results are directly filtered without sending them via the network. Correctness is guaranteed as no other data source can contribute to the group of triple patterns with further information.

Consider the query given in Listing 2. The first two triple patterns form an exclusive group, since DrugBank is the single relevant source for both of the triple patterns. Similarly, the third and fourth triple pattern form another exclusive group for KEGG data source. Thus the first two triple patterns can be directly executed by DrugBank and the next two triple patterns can be executed by KEGG.

Our SPARQL 1.0 to SPARQL 1.1 query re-write makes use of the exclusive groups, SPARQL SERVICE, and SPARQL UNION clauses as follow: (1) identify exclusive groups from the results of the sources selection, (2) group each exclusive group into a separate SPARQL SERVICE, and (3) write a separate SPARQL SERVICE clause for non-exclusive group triple patterns for each of the relevant source and UNION the triple pattern results from each relevant source by using SPARQL UNION clause.

A SPARQL 1.1 query in Listing 3 is a re-write of the SPARQL 1.0 query given in Listing 2. The first exclusive group of triple patterns (i.e., triple patterns 1–2) are grouped into DrugBank SERVICE while the second exclusive group of triple patterns (i.e., triple patterns 3–4) are grouped into KEGG SERVICE. Since both KEGG and ChEBI are the relevant data sources for the last triple pattern, a separated SPARQL SERVICE is used for each of the data source and the results are union-ed using SPARQL UNION clause. The final SPARQL 1.1 query is then directly executed using Jena API.

### Experimental setup

The experiments were performed on computers running federation engines having as a system setup: 2.53 GHz i5 processor, 8GB RAM and 320GB hard disk. Data sets are arranged in a fashion that those having federated queries are resided on different systems. For the system with Java implementation, we used Eclipse as the default setting, i.e., Java Virtual Machine (JVM) initial memory allocation pool (Xms) size of 128.53MB and the maximum memory allocation pool (Xmx) size of 2057.30MB. The permanent generation (MaxPermSize) which defines the memory allocated to keep compiled class files has been set to 21.75MB as the default size. We used FedX^16^ version 2012 as one of the federation engines in Java. In order to reduce the network latency we used a dedicated local network. We conducted our experiments on local instances of Virtuoso.

We used the most recent virtuoso version 07.10.3207 for SPARQL endpoints having specifications such as number of buffers 34,000, maximum dirty buffers 250,000, number of server threads 20, result set maximum rows 100,00, and maximum SPARQL endpoint query execution time of 60 seconds. A separate physical virtuoso server was created for 5 data sets i.e., Sider, Medicare, Dailymed, Diseasome and LinkedCT.

The remaining 5 datset virtuoso instances i.e., LinkedTCGA, Drugbank, Kegg, Chebi and Affymetrix were carried out from bigrdfbench ^17^ The specification of the machines hosting the virtuoso SPARQL endpoints used in evaluations is given in Table 1. In order to get the maximum results the query timeout was set to be ’0’ and we run each query once.

**Table 1:** Hardware statistics

Endpoint name	Operating system	CPU(GHz)	RAM	Hard disk
Chebi	Window 7 Professional Service Pack 1 64 bits	2.90, i7	8GB	148GB
LinkedTCGA	Window 7 Professional Service Pack 1 64 bits	2.90, i7	8GB	148GB
Sider	Window 7 Professional Service Pack 1 64 bits	2.90, i7	8GB	148GB
Dailymed	Window 7 Professional Service Pack 1 64 bits	2.26, 2 Duo	4GB	148GB
Medicare	Window 7 Professional Service Pack 1 64 bits	2.26, 2 Duo	4GB	148GB
LinkedCT	Window 7 Professional Service Pack 1 64 bits	2.53, i5	4GB	297GB
Diseasome	Window 7 Professional Service Pack 1 64 bits	2.53, i5	4GB	297GB
Affymetrix	Ubuntu 14.04 LTS 64 bits	1.80, i5	8GB	256GB
Drugbank	Ubuntu 14.04 LTS 64 bits	1.80, i5	8GB	256GB
Kegg	Ubuntu 14.04 LTS 64 bits	2.53, i5	8GB	320GB

## BioFed salient features

In this section we explain the key features of BioFed. BioFed uses Apache Jena and thus fully supports SPARQL 1.1.

### Provenance

Earlier on we determined that we needed to understand which SPARQL endpoints were responding to queries and with how much data. The approach is to record the start and end times of the SPARQL results, as well as count the number of items returned. This data is written to the standard system logging framework using the system generated query id to identify the source query. In addition to logging the data to the standard framework a custom log4j Filter was created that intercepts the log messages generated by the query.

To achieve this three (3) components were developed:

1. A Jena SPARQL extension function (CounterFunction) to record start, end and elapsed times as well as number of triples returned for each Service endpoint.
2. A Jena QueryIterator (TracingIterator) that logs the start and end of the query as well as reporting the results of each of the enclosed extension functions.
3. A thread name filter (ThreadNameFilter) that filters the logging entries for a specific query.

When a query is received it is rewritten into a series of SPARQL SERVICE calls. Each of these are wrapped in a CounterFunction. The entire query execution is wrapped in a TracingIterator. Before execution is commenced a logging listener is attached to the logging framework and a ThreadNameFilter is attached to limit the collection to only entries from the query. At the end of query execution, the logging result is stored in a temporary cache where the user can request it via a REST web service call. Data was only retained for 10 min or until the next query was executed for the same the user. Information provided included the total execution time, the number of triples returned and any error indications including whether an endpoint was down.

### Data access

BioFed provides query access to the endpoint availability data via the EndpointData graph. Once selected SPARQL queries can retrieve the endpoint data including latency, up or down status, whether or not the data for the endpoint has been initialised, when it was last checked and what the endpoint URL is.

BioFed uses the ARDI approach to identify and process the data sets from multiple endpoints and prioritises data from the endpoints with lowest latency responses. Therefore, BioFed reduces duplicates for identical specifications of data triples. Unfortunately, this still leaves a number of problems unresolved, such as URI mismatches for – seemingly – identical entities, i.e. in the case of the reshaping of URIs upon reuse of content from public resources. Similar problems arise, if separate resources do not share namespaces but make reference to semantically identical classes or types. BioFed does not perform duplicates detection within single data sets.

BioFed does not directly support a no-blocking operator. It does preemptive checking to ensure that an endpoint is available. In the case where multiple endpoints provide the same data it selects the endpoint which responds with lowest latency. In addition, the underlying Jena framework provides parameters for the abortion of queries and then returns partial results, if the endpoint does not finalise the query.

BioFed would not return complete results under the following conditions (apart from network or hardware failures). First, ARDI can be out of date just like any other index, since it is affected from data being added to an endpoint that then cannot be retrieved, and from data being removed and thus leading to partial results due to conditions of the SPARQL query will not being met. Second, an endpoint could be non-responsive thus not producing results.

### BioFed web interface

The web interface provides the ability to directly enter a SPARQL query into an input box or to use the *Standard Query Builder*. The users are provided the option of viewing the results directly or downloading the results as a file in one of six (6) formats including Text, Comma Separated Values (CSV), Tab Separated Values (TSV), JavaScript Object Notation (JSON), Turtle and Extensible Markup Language (XML).

The default or standard query builder is an interface that provides a list of topics. When one topic is selected all the attributes of that topic are listed. This set of topics known as Query Elements (Qe), are the list of concepts from different SPARQL endpoints and can be replaced by any other set of concepts define in any context e.g., Protein Protein Interaction or directly from other SPARQL endpoints. The user selects the attribute and enters the desired value either as a variable or a literal. The requisite lines are then added to the query input box. Multiple selections may be added to the query after which it can be edited. The steps for formulating SPARQL query are listed in the User Guide available at: http://srvgal78.deri.ie/BioFed/.

As mentioned earlier, the current BioFed interface supports making of basic SPARQL queries. This does not mean that BioFed supports only these queries but supports a full range of simple and complex queries sent to public SPARQL endpoints, available at the time of the query and catalogued in ARDI. This includes queries listed in Listing 4–23.

## Results and discussion

### Data sets for the experimental setup

Our experiments are based on 10 real-world data sets. All the data sets were collected from life sciences domains as BioFed is a query engine for life sciences. We began by selecting all three real world data sets from Fedbench [36] namely Drugbank^18^ a knowledge base containing information pertaining to drugs, their composition and their interactions with other drugs, Chebi- the Chemical Entities of biological Interest^19^, Kegg Kyoto Encyclopedia of Genes and Genomes (KEGG)^20^ which contains further information about chemical compounds and reactions with a focus on information relevant for geneticists.

We added one sub-data set from Cancer Genome Atlas^21^ (TCGA) presented in [3], along with the Affymetrix^22^ data set that contains the probesets used in the Affymetrix microarrays. The subset from TCGA known as TCGA-A contain methylation, exon. Moreover, Linked TCGA-A has a large number of links to Affymetrix, which we added to the list of our data sets.

Apart from the aforementioned selected data sets, five other data sets were chosen that had connectivity with the existing ones that enabled us to include real federated queries. These data sets include SIDER^23^ – which contains information on marketed drugs and their adverse effects, Diseasome^24^ – which publishes a network of 4,300 disorders and disease genes linked by known disorder-gene associations for exploring all known phenotype and disease gene associations, indicating the common genetic origin of many diseases., Dailymed^25^ – provides information about marketed drugs including the chemical structure of the compound, its therapeutic purpose, its clinical pharmacology, indication and usage, warnings, precautions, contraindications, adverse reactions, over dosage etc., LinkedCT^26^ – publishes clinical Trials and Medicare^27^.

**Fig. 2:**
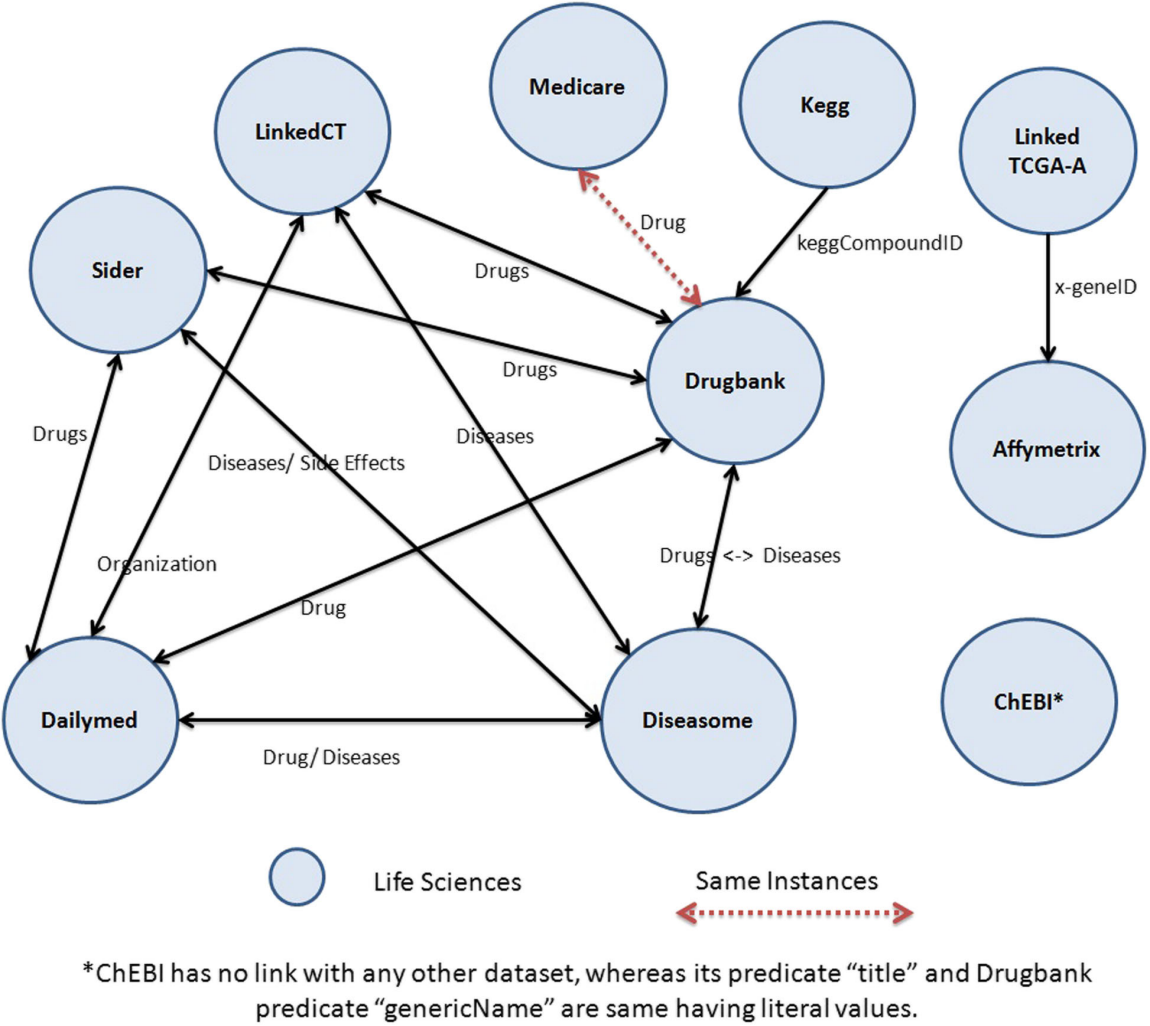
Datasets connectivity. Connectivity overview of some Life science data sets through classes/properties, used in experimental setup

Figure 2 shows the topology of all 10 data sets selected for BioFed while some other basic statistics like the total number of triples, the number of resources, predicates and objects, as well as the number of classes and the number of links can be found in Table 2. It is important to note that ChEBI has no link with any other data set. However, its predicate *“title”* and DrugBank’s predicate *“genericName”* display the same literal values. Similarly, the Linked TCGA-A predicate *“drug_name”* and DrugBank’s *“genericName”* display the same values.

**Table 2:** Dataset statistics

Dataset	Triples	Subjects	Predicates	Objects	Classes	Structuredness
Chebi	4772706	50477	28	772138	1	0.340
DrugBank	517023	19693	119	276142	8	0.726
Kegg	1090830	34260	21	939258	4	0.919
Affymetrix	44207146	1421763	105	13240270	3	0.506
Dailymed	162972	10015	28	67782	6	0.663
Diseasome	72445	8152	19	27704	4	0.543
Sider	101542	2674	11	29410	4	0.924
Medicare	44500	6825	6	23308	3	0.843
LinkedCT	9804652	981880	90	3808369	13	0.840
Linked TCGA-A	35329868	5782962	383	8329393	23	0.998
Total	96103684	8318701	810	27513774	69	-

As defined by [37] the *data sets* used in the federated SPARQL environment should complement each other in terms of the total number of triples, number of classes, number of resources, number of properties, number of objects, average properties and instances per class, etc. Duan et al. [37] combine these features into a single composite metric called *structuredness or cohesion*. For a given data set, the structuredness value covers the range [0,1] with 0 means exposing less structured and 1 highly structured data sets. A federated SPARQL query benchmark should comprise data sets of varying structuredness values so is the case of our selected data sets (Table 2).

### Query

BioFed is able to support and federate any SPARQL query issued to those publicly available points that are catalogued in through ARDI and that are available at the time of query. Our evaluation comprises a total of 20 queries for *SPARQL endpoint federation approaches*. These queries are divided into two different types: the 10 simple queries (see listings S1–S10, 4–13) and 10 complex queries (see listings C1–C10, 14–23). Table 3 shows key features and statistics of these queries.

**Table 3:** Comparison of the queries in terms of basic graph patterns *#BGPs*, Triple Patterns *#TP*, total vertices *TVs*, join vertices *JVs*, join vertices to total vertices Ratio *R* and mean join vertices degree *D* per query

Query	QueryType	#BGPs	#TP	TVs	JVs	R	D	SPARQL Clauses
SQ1	Simple	2	4	10	2	0.20	2.0	UNION
SQ2	Simple	1	7	15	4	0.266	2.5	X
SQ3	Simple	1	6	13	4	0.307	2.250	X
SQ4	Simple	1	5	11	3	0.272	2.333	X
SQ5	Simple	2	5	12	3	0.250	2.0	OPTIONAL
SQ6	Simple	1	3	7	2	0.285	2.0	X
SQ7	Simple	1	4	9	3	0.333	2.0	X
SQ8	Simple	1	3	7	2	0.285	2.0	X
SQ9	Simple	1	8	14	2	0.117	4.5	DISTINCT
SQ10	Simple	1	8	17	2	0.117	4.5	DISTINCT
CQ1	Complex	2	8	18	4	0.222	2.5	DISTINCT, OPTIONAL, FILTER
CQ2	Complex	2	8	19	4	0.210	2.25	OPTIONAL, FILTER
CQ3	Complex	1	10	19	4	0.210	3.75	DISTINCT, FILTER, REGEX
CQ4	Complex	1	6	13	4	0.307	2.25	X
CQ5	Complex	2	10	22	3	0.136	3.666	OPTIONAL
CQ6	Complex	2	12	24	6	0.25	3.0	OPTIONAL
CQ7	Complex	1	8	17	4	0.235	2.75	X
CQ8	Complex	1	6	13	2	0.153	3.5	X
CQ9	Complex	1	9	19	5	0.263	2.6	FILTER
CQ10	Complex	2	9	20	3	0.15	3.333	OPTIONAL

Some of the simple queries e.g., SQ2, SQ3, SQ4, SQ5 are taken from the existing benchmark Fedbench [36]. To the best of our knowledge, none of the existing benchmarks can be considered for selecting the full range of queries. Fedbench provides queries not limited to the life sciences domain but also cover Cross Domain, SP2B and Linked Data, not relevant for BioFed. Atsuko et al. provides Bio Benchmark [38], which does not define federated queries and therefore is not relevant for BioFed. Hence the rest of the simple queries and 10 complex queries CQ1-CQ10 were created in close collaboration with domain experts.

#### Types of queries

Simple queries comprise the smallest number of triple patterns, which range from 2 to 8. These queries require retrieving data from 2 to 5 data sources (ref: listing: 4–13). Moreover, these queries only use a subset of the SPARQL clauses as shown in Table 3, and do not expose constraints on the queries via LIMIT, REGEX, DISTINCT and ORDER BY clauses. Their query execution time is small. By contrast, complex queries (14–23) have no restrictions on the number of used triple patterns nor on the SPARQL clause features.

Gorlitz et al. [39] and Aluc et al. [40] propose different *query characteristics* for benchmarking federated queries. These include: *number of basic graph patterns (BGP* ^15^ *)*, *number of triple patterns*, *number of vertices*, *number of join vertices*, *mean join vertex degree*,and use of different *SPARQL clauses* (e.g., LIMIT, OPTIONAL, ORDER BY, FILTER, DISTINCT, UNION, REGEX). A vertex represents the subject, predicate, or object of a triple pattern, and can be any of a URI, literal, blank node, or a variable [40]. The number of join vertices represents the number of vertices that are the subject, predicate or object of multiple triple patterns in a BGP. A join vertex degree of a join vertex x ∈ BGP is the number of triple patterns in the same BGP whose subject, predicate or object is x.

Consider the query given in listing: 9, the number of BGPs is 1, the number of triple patterns is 3, the number of vertices is 7 (i.e., ?drug, drugbank:molecularWeightAverage, ?weight, drugbank:possibleDiseaseTarget, ?disease, diseasome:name, and ?name), the number of join vertices is 2 (i.e., ?drug, ?disease), the join vertex to total vertex ratio is 0.285 (i.e., 2/7), mean join vertex degree is 2.0 (i.e., both join vertices ?drug and ?disease are used in two triple patterns, thus each has a degree of 2), and no aforementioned SPARQL clause is used the query. We considered all these *SPARQL queries features* while selecting our queries as shown in Table 3.

### Performance metrics

For BioFed, the TTPWSS for the query given in listing: 2 is 6 (i.e., 1+1+1+1+2).

We have selected five performance metrics in our evaluation:

- Total triple pattern-wise sources selected (TTPWSS)
- Number of SPARQL ASK requests used during the source selection
- Source selection time
- Overall Query execution time
- Result set completeness

Previous works [1, 14, 41] show that these are the key metrics for the performance evaluation of the SPARQL endpoint federation systems. For example, an over estimation of the TTPWSS results in extra network traffic in form of irrelevant intermediate results, thus increasing the overall query execution time. The time consumed by the number of SPARQL ASK requests used during the source selection and the corresponding source selection time is directly added to the over all query execution time. Two federation systems can only be compared to each other if they retrieve the same number of results for a given SPARQL query. Furthermore, previous work [1] shows that the SPARQL endpoint federation engine can miss results due to an out-of-date index, SPARQL endpoint restrictions, join implementation etc.

Based on the above metrics and queries discussed in Section “Query”, we compared FedX [11] (the fastest state-of-the-art federation engine [1]) with BioFed and present the results in next section.

### Evaluation results

#### Efficiency of source selection

We define source selection efficiency in terms of (a) total number of triple-wise sources selected (#TP), (b) SPARQL ASK requests used (#AR; to obtain (a)), and (c) the source selection time (SST). Table 4 represents the results collected based on these three metrics. Before going into the details, it is important to mention that FedX makes use of the cache to store recent SPARQL ASK requests used during the source selection. In this section, we presents the results for FedX(cold), i.e., when cache is completely empty. For FedX(100%cached), the number of SPARQL ASK requests used the during selection will be zero.

**Table 4:** Comparison of the source selection in terms of number of ASK *#AR*, total triple pattern-wise sources selected *#TP*, source selection time *SST* in msec and total number of results retrieved *#R* per query. *T/A* = Total/Avg., where Total is for #TP, #AR, and Avg. is for #SST

	FedX(cold)				BioFed			
Query	#AR	#TP	SST	#R	#AR	#TP	SST	#R
SQ1	40	4	3374	5146	0	4	1061	5146
SQ2	70	7	3513	3	20	7	386	3
SQ3	60	8	3194	393	10	8	280	403
SQ4	50	7	3234	6	20	7	6255	28
SQ5	50	6	3289	1620	0	6	849	1620
SQ6	30	3	3281	8120	0	3	47	8120
SQ7	40	19	4088	27	0	19	3804	27
SQ8	30	2	3587	0	0	2	165	0
SQ9	80	11	3218	-	10	11	297	-
SQ10	80	11	3234	-	10	11	268	-
T/A	530	78	3401	-	70	78	1341	-
CQ1	80	9	3354	-	10	9	249	-
CQ2	80	9	3242	4	10	9	2238	4
CQ3	100	28	3148	7	20	28	1743	-
CQ4	60	12	3136	133986	0	12	1967	134025
CQ5	100	16	3751	2940	10	16	1122	2940
CQ6	120	18	4675	4781	10	18	694	4781
CQ7	80	8	3283	372	0	8	713	372
CQ8	60	6	9621	21	20	6	560	21
CQ9	90	9	7112	-	10	9	195	-
CQ10	90	15	93852	22888	0	15	1345	63948
T/A	860	104	135174	-	90	104	1082	-
Net T/A	1390	182	69287	-	160	182	1211	-

As an overall source selection evaluation, BioFed is more efficient than FedX(cold) in terms of the number of SPARQL ASK requests consumed (1390 vs. 160) and the source selection time (69287 ms vs. 1211 ms). While in terms of total triple pattern-wise sources selected, both of the systems exactly select the same number of sources for all the benchmark queries. The reason for BioFed’s source selection efficiency is the use of the ARDI and the two step source selection, i.e., first selected the relevant sources using the ARDI and then prune the selected sources using SPARQL ASK request (ref. Section “Source selection”). On the other hand, FedX(cold)’s complete source selection is based on SPARQL ASK requests, i.e., sends a SPARQL ASK request to all of the data sources for all query triple patterns. Thus for a given SPARQL query, the total number of ASK requests used by FedX(cold) is the product of the total number of data sources and the total number of triple patterns in the query. As SPARQL ASK requests are alike to SPARQL SELECT requests without result production, therefore, the number of SPARQL ASK correlates to the source selection time.

The reason for the exact same number of total triple pattern-wise sources selected is that both make use of the SPARQL ASK requests when either subject or object of the triple pattern is bound. Since BioFed’s ARDI stores all the distinct predicates for each of the data sources, for triple patterns with only bound predicates, it results in optimal data source selection.

Fedx doesn’t check the source availability i.e., whether the source is up and ruining, and treats all the selected source as live, up and running and makes the federated query based on these. Therefore, it was noticed that some time query adds some dead sources and doesn’t reply because of hang on issues. However, BioFed facilitates the pre source availability test and makes federated query only for those sources which are up and running. Furthermore, we introduced the use of SPARQL ASK requests combined with SSGM (Star Shaped Group Multiple Endpoints) [41] to reduce the selected source which shortened the SPARQL federated query.

#### Result-set completeness

Two systems can only be compared to each other if they produce the same results for a given SPARQL query. We have observed both of the systems cannot guarantee result-set completeness.

Table 5 shows the set of queries for which one of the systems results in incomplete results. The values inside bracket, e.g., SQ3(393) shows the actual query results. There can be a number of reasons, e.g., network conditions, use of out-of-date date index, SPARQL endpoints restrictions (e.g., maximum result-set size of 10000), incomplete source selection, and join implementation etc. for which a system may result in incomplete results. However, in our case, we used a dedicated local area network, always up-to-date index’s, and no endpoint restrictions. A possible reason for the result-set incompleteness might be optimised execution plan generation and the join order implementations.

**Table 5:** Result set completeness and correctness: Table 5 below represents the result completeness and correctness

System	SQ3 (393)	SQ4 (28)	CQ3(7)	CQ4 (133986)	CQ10(22888)
FedX	393	6	7	133986	22888
BioFed	403	28	-	134025	63948

The values in brackets tells the actual data size. The symbol *-* means either the query didn’t return the complete results or unlimited query execution time

#### Query execution time

**Fig. 3:**
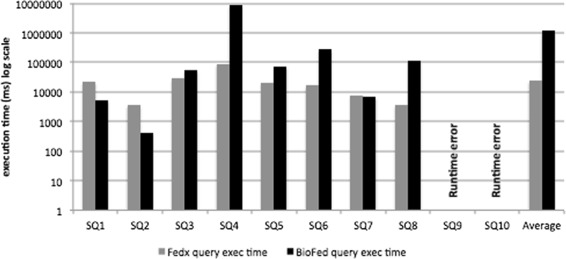
Query execution time for simple category queries. Comparison of simple queries execution time run on FedX and BioFed

**Fig. 4:**
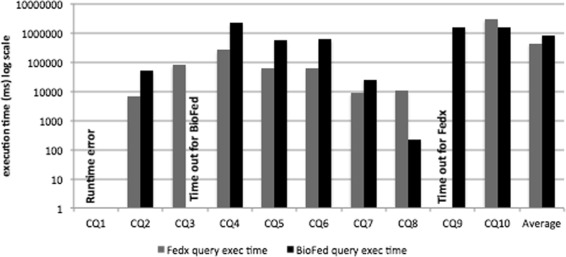
Query execution time for complex category queries. Comparison of complex queries execution time run on FedX and BioFed

Query execution time is considered to be one of the key metrics for the performance evaluation of the federated engines. Figures 3 and 4 show a comparison of the query execution time for simple and complex category queries, respectively. As an overall query execution time evaluation, FedX performs better than BioFed in 5 out of 8 comparable queries in the category of simple queries (for two queries resulted into runtime errors for both systems) and 5 out of 7 comparable queries in the category complex queries.

There are two main reasons for BioFed’s slightly lower performance in a few queries: (1) the source selection becomes less efficient for queries containing common predicates, e.g., rdf:type, rdfs:label, owl:sameAs etc. which results in big SPARQL 1.1 queries, thus taking more time to be executed on top of the Jena API and (2) some extra time has to be spent for the collectiion and maintainance of the provenance information, which is not provided by FedX as a feature. We believe that the replacement of BioFed’s source selection with more efficient *join-aware* source selection as in HiBISCuS [14], we would greatly improve the query execution time for queries containing more common predicates.

## Conclusions

In this paper we presented BioFed, a user friendly system for federated SPARQL query processes based on real biological data addressing meaningful biological queries and using the large-scale and complex life science sources as a challenging real life scenario. Its Web based interface facilitates query generation which would pose major difficulties for biological scientists otherwise. Currently the interface supports only basic query building using Qe provided by different SPARQL endpoints and in future we aim to provide an interface that can be able to formulate complex SPARQL queries. Moreover as a future work, we also want to provide an intuitive visual interface for query formulation and execution as provided by project like FedViz [42].

BioFed uses ARDI – a dynamically generated catalogue for all publicly available SPARQL endpoints relevant to the scientific domain. We presented two different cateogires and sets of queries and compared the query exeuction in BioFed with the state-of-the-art system FedX. Our results suggest that our system is superior in terms of time taken to retrieve the required information. Specific queries remained un-answered for both systems.

To the best of our knowledge, the important aspects associated with biological data, like provenance, is implemented for the first time in BioFed. In future, we will focus to improve the overall performance of BioFed by efficient source selection using the join-aware TPWSS as implemented by HiBISCuS.

We believe that the proposed system can greatly help researchers in the biomedical domain to carry out their research by effectively retrieving relevant life science data. As the amount and diversity of biomedical data exceeds the ability of local resources to handle its retrieval and parsing, BioFed, facilitates federation over diverse resources.

## Listings







### Simple Federated SPARQL Queries





















### Complex Federated SPARQL Queries





















## Endnotes

^1^ http://stats.lod2.eu/(l.a.: 25 Feb 2017)

^2^ http://linkeddatacatalog.dws.informatik.uni-mannheim.de/state/(l.a.: 25 Feb 2017)

^3^ http://bio2rdf.org/(l.a.: 25 Feb 2017)

^4^ http://linkedlifedata.com/(l.a.: 25 Feb 2017)

^5^ http://neurocommons.org/page/Main_Page(l.a.: 25 Feb 2017)

^6^ http://www.w3.org/TR/hcls-kb/(l.a.: 25th Feb 2017)

^7^ http://www.w3.org/wiki/HCLSIG/LODD(l.a.: 25 Feb 2017)

^8^ http://swobjects.org/(l.a.: 25 Feb 2017)

^9^ https://jena.apache.org/(l.a.: 25 Feb 2017)

^10^ http://dotnetrdf.org/(l.a.: 25 Feb 2017)

^11^ http://goo.gl/ZLbLzq(l.a.: 25 Feb 2017)

^12^ http://wiki.ckan.org/Main_Page(l.a.: 25 Feb 2017)

^13^ http://kegg.bio2rdf.org/sparql(l.a.: 25 Feb 2017)

^14^ Jena API: http://jena.apache.org/documentation/query/(l.a.: 25 Feb 2017)

^15^ Basic graph patterns: http://www.w3.org/TR/sparql11-query/#BasicGraphPatterns(l.a.: 25 Feb 2017)

^16^ http://www.fluidops.com/FedX/(l.a.: 25 Feb 2017)

^17^ https://code.google.com/p/bigrdfbench/(l.a.: 25 Feb 2017)

^18^ http://www.drugbank.ca/(l.a.: 25 Feb 2017)

^19^ https://www.ebi.ac.uk/chebi/(l.a.: 25th Feb 2017)

^20^ http://www.genome.jp/kegg/(l.a.: 25th Feb 2017)

^21^ http://cancergenome.nih.gov/(l.a.: 25 Feb 2017)

^22^ http://www.affymetrix.com/(l.a.: 25 Feb 2017)

^23^ http://wifo5-03.informatik.uni-mannheim.de/sider/(l.a.: 25 Feb 2017)

^24^ http://wifo5-03.informatik.uni-mannheim.de/diseasome/(l.a.: 25 Feb 2017)

^25^ http://dailymed.nlm.nih.gov/dailymed/index.cfm(l.a.: 25 Feb 2017)

^26^ http://linkedct.org/(l.a.: 25 Feb 2017)

^27^ http://wifo5-03.informatik.uni-mannheim.de/medicare/(l.a.: 25 Feb 2017)

## Abbreviations

BGP: Base graph pattern
CSV: Comma separated values
DSQE: Domain specific query engine: JSON: JavaScript object notation
LOD: Linked open data
LS: Life science
LS-LOD: Life sciences linked open data
QE: Query engine: Qe: Query elements
TPWSS: Triple pattern-wise source selection
TSV: Tab seperated values
VoID: Vocabulary of interlinked datasets
XML: Extensible markup language
